# Self-administration of rozanolixizumab via manual push and infusion pump methods in patients with generalised myasthenia gravis: a randomised, phase 3, open-label, crossover study

**DOI:** 10.1007/s00415-025-13420-6

**Published:** 2025-10-11

**Authors:** Vera Bril, Carlo Antozzi, Tomasz Berkowicz, Artur Drużdż, Rachana K. Gandhi Mehta, Zabeen K. Mahuwala, Jana Zschüntzsch, Marion Boehnlein, Virginie Kerbusch, Andreea Lavrov, Mark Morris, Puneet Singh, M. Isabel Leite

**Affiliations:** 1 Ellen and Martin Prosserman Centre for Neuromuscular Diseases, Toronto General Hospital, University of Toronto, Toronto, ON Canada; 2 Immunotherapy and Apheresis Unit and Neuroimmunology and Muscle Pathology Unit, Fondazione Istituto di Ricovero e Cura a Carattere Scientifico, Istituto Neurologico Carlo Besta, Milan, Italy; 3 Miejskie Centrum Medyczne Jonscher im. dr. Karola Jonschera w Łodzi, Łodź, Poland; 4 Department of Neurology, Municipal Hospital, Poznań, Poland; 5 Department of Neurology, Wake Forest University School of Medicine, Winston-Salem, NC USA; 6 Department of Neuromuscular Medicine, Epilepsy and Clinical Neurophysiology, University of Kentucky, Lexington, KY USA; 7 Department of Neurology, University Medical Center Göttingen, Göttingen, Germany; 8 UCB, Monheim, Germany; 9 PharmAspire BV, Wijchen, The Netherlands; 10 UCB, Slough, UK; 11 Nuffield Department of Clinical Neurosciences, University of Oxford, Oxford, UK

**Keywords:** Generalised myasthenia gravis, Rozanolixizumab, Self-administration, Infusion pump, Manual push, Patient preference

## Abstract

**Background:**

The phase 3, open-label, randomised, crossover MG0020 study investigated rozanolixizumab self-administration, efficacy, and safety in patients with generalised myasthenia gravis (gMG) using infusion pump and manual push methods.

**Methods:**

Adults with gMG received once-weekly rozanolixizumab for 18 weeks, comprising a 6-week Training Period and two 6-week Self-Administration Periods where patients were randomised 1:1 to Sequence 1 (infusion pump then manual push) or Sequence 2 (manual push then infusion pump). The primary endpoint was successful rozanolixizumab self-administration (choosing correct infusion site, administering subcutaneously, delivering intended dose), evaluated by a healthcare professional (HCP) at weeks 12 and 18. Secondary endpoints included treatment-emergent adverse events (TEAEs). Additional endpoints included change from baseline in total immunoglobulin G (IgG) and Myasthenia Gravis Activities of Daily Living (MG-ADL) score, and patients’ administration method preference.

**Results:**

Sixty-two patients received treatment; 55 were randomised (Sequence 1: *n* = 28; Sequence 2: *n* = 27). The self-administration success rate was 100% with both methods. Decreases from baseline in IgG and MG-ADL score were maintained during self-administration with both methods. TEAEs occurred in 47/62 (75.8%) patients; most events (161/165 [97.6%]) were mild or moderate. Incidence was comparable for both methods. Most patients (35/55 [63.6%]) preferred self-administration to HCP administration (5/55 [9.1%]); more preferred manual push (25/55 [45.5%]) to infusion pump (17/55 [30.9%]).

**Conclusions:**

All patients successfully self-administered rozanolixizumab; more patients preferred manual push. Efficacy and safety were consistent with the known HCP-administered profile. These results support rozanolixizumab self-administration and manual push administration in patients with gMG.

*Trial registration*: NCT05681715 (registered 4 January 2023).

**Supplementary Information:**

The online version contains supplementary material available at 10.1007/s00415-025-13420-6.

## Introduction

Myasthenia gravis (MG) is a chronic autoimmune disorder affecting the neuromuscular junction, characterised by fluctuating fatigable muscle weakness [1, 2]. Several mechanisms are believed to drive MG pathogenesis, including pathogenic immunoglobulin G (IgG) autoantibodies, which can impair synaptic transmission at the neuromuscular junction [2]. In recent years, MG treatment options have expanded beyond oral first-line therapies (corticosteroids, immunosuppressants, and pyridostigmine) and intravenous (IV) infusions to include subcutaneous (SC) administration [3, 4]. This shift has been driven by advances, such as fixed-dose SC formulations, technologies that facilitate the injection of dosing volumes > 5 mL, and devices that enable self-administration outside the hospital setting [5]. SC immunoglobulins (SCIg) are one of the most common SC therapies, used to treat both antibody deficiencies and autoimmune diseases [6, 7]. Many monoclonal antibodies can be administered subcutaneously in the treatment of autoimmune diseases [8–10]. Following education and training, SC treatment may be self-administered by the patient or a caregiver if approved [11]. The success of SC self-administration has been well documented in patients with primary immunodeficiency (PID) and chronic autoimmune diseases, such as chronic inflammatory demyelinating polyneuropathy [11–14]. Self-administered treatment options have also been approved for use in patients with generalised MG (gMG) [15, 16]. In a meta-analysis evaluating treatment administration preferences in patients with autoimmune disease, including MG, 83% of patients preferred SC over IV infusions. In addition, 84% preferred at-home administration compared with treatment administration in a hospital setting [11]. SC self-administration at home offers many advantages for patients over IV administration, such as high satisfaction, a greater sense of control, improved health-related quality of life (HRQoL) and increased convenience and independence, by eliminating the need for frequent and time-consuming infusion visits [11, 17–19].

Infusion pump and manual push methods are two potential options that allow self-administration of subcutaneous infusions by patients. Whilst both methods provide independence from administration by healthcare professionals (HCPs), the manual push method is simpler, more cost-effective, requires less equipment, and is expected to reduce infusion times [20, 21]. Studies of self-administration of SCIg in patients with PID demonstrated faster infusion rates, with comparable safety [20] and greater patient preference for the manual push method compared with the infusion pump [22].

Rozanolixizumab is a humanised IgG4 monoclonal antibody neonatal Fc receptor (FcRn) blocker that targets the IgG-binding region of the neonatal FcRn to inhibit IgG recycling and reduce levels of circulating IgG autoantibodies [23, 24]. In the randomised, double-blind, placebo-controlled, phase 3 MycarinG study (NCT03971422; EudraCT 2019-000968-18), one 6-week cycle of once-weekly SC rozanolixizumab infusions was generally well tolerated and demonstrated clinically meaningful improvements in MG-specific outcomes in patients with anti-acetylcholine receptor (AChR) or anti-muscle-specific kinase (MuSK) antibody-positive (Ab+) gMG [24]. In pooled analyses of data from MycarinG and the open-label extension studies MG0004 (NCT04124965; EudraCT 2019-000969-21) and MG0007 (interim data; NCT04650854; EudraCT 2020–003230-20), the long-term safety, tolerability, and efficacy of rozanolixizumab were further demonstrated [25, 26].

Following the MycarinG study, rozanolixizumab was approved for SC administration by HCPs once weekly for 6-week cycles, using a programmable infusion pump [27–29]. Given the chronic nature and high burden of gMG symptoms and associated care [30], there was interest in providing an option for self-administration of rozanolixizumab to allow patients and caregivers to administer treatment in accordance with their individual needs and preferences. Here, we report findings from the phase 3 MG0020 study that evaluated patients’ ability to successfully self-administer rozanolixizumab using the manual push and infusion pump self-administration methods. A plain language summary of this analysis is available in Online Resource 1.

## Methods

### Study design and patients

**Fig. 1:**
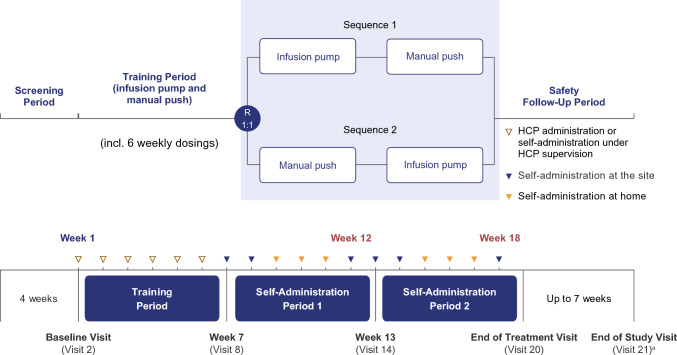
Study design. ^a^Patients completing all treatment periods, including the End of Treatment Visit, and moving on to either a post-study access programme or commercially available rozanolixizumab during the Safety Follow-Up Period underwent an earlier End of Study Visit prior to this move. HCP, healthcare professional; R, randomisation.

MG0020 (NCT05681715; EudraCT 2022–003870-21) was a phase 3, randomised, open-label, 2-period, 2-sequence crossover study investigating the successful self-administration, efficacy and safety of rozanolixizumab in patients with gMG (Fig. 1). Patients received rozanolixizumab (fixed dosing [420 mg for body weight 35–< 50 kg; 560 mg for body weight ≥ 50 kg] or weight-tiered dosing of 7 mg/kg [Japanese participants only]) once weekly for 18 consecutive weeks, consisting of a 6-week self-administration Training Period followed by two 6-week Self-Administration Periods. This study included a Safety Follow-Up Period of up to 7 weeks. At the end of the Training Period (week 7) and following the investigator’s confirmation of eligibility to perform self-administration, interactive response technology was used to randomise patients based on a predetermined randomisation schedule. Patients were randomised 1:1 to self-administer rozanolixizumab using the infusion pump method first followed by the manual push method (Sequence 1), or manual push first followed by the infusion pump method (Sequence 2).

Eligible patients were ≥ 18 years old with a documented diagnosis of gMG, serum total IgG level of 5.5–16.0 g/L and body weight ≥ 35 kg. Key exclusion criteria were clinically relevant active infection or history of serious infection within 6 weeks prior to the baseline visit, severe oropharyngeal or respiratory weakness, myasthenic crisis or impending crisis, absolute neutrophil count < 1500 cells/mm^3^, active malignant neoplastic disease, and history of malignant neoplastic disease within 5 years of screening. Treatment with IVIg, SCIg, and plasma exchange (PLEX) within 2 weeks before the baseline visit was not permitted. Concomitant IVIg, SCIg, and PLEX could be administered as rescue therapy at the investigators’ discretion; however, patients who received rescue therapy were withdrawn from the study. The protocol was approved by an Institutional Review Board/Independent Ethics Committee and all patients provided written informed consent.

### Outcomes

The primary endpoint was successful self-administration of rozanolixizumab by infusion pump and manual push methods, evaluated by an HCP at the end of each 6-week Self-Administration Period (weeks 12 and 18), and defined as choosing the correct infusion site, administering subcutaneously and delivering the intended dose. Secondary endpoints were the occurrence of treatment-emergent adverse events (TEAEs) after infusion pump or manual push self-administration from week 1 up to the End of Study (EOS) Visit, occurrence of local site reactions up to 24 h after each administration during the Training Period and two Self-Administration Periods, and occurrence of medication errors associated with adverse reactions during the two Self-Administration Periods. Additional endpoints were the occurrence of TEAEs leading to permanent withdrawal of rozanolixizumab, successful self-administration of rozanolixizumab via infusion pump and manual push methods at each of the three home administration visits during Self-Administration Periods 1 and 2, respectively, total IgG levels over time, incidence of anti-drug antibodies (ADAs), change from baseline in Myasthenia Gravis Activities of Daily Living (MG-ADL) score, and patient preference for self-administration versus HCP administration and for infusion pump versus manual push administration. Patient treatment satisfaction was assessed pre- and post-treatment at week 1 and at weeks 11 and 17, using an infusion adaptation of the Self-Injection Assessment Questionnaire (SIAQ) [31]. Domain scores were assessed on a 0–10 scale, where higher scores indicate a more positive experience.

### Statistical analysis

The primary outcome was analysed in the full analysis set, comprising all patients who were included in the safety set, were randomised, completed both Self-Administration Periods, performed self-administration at both weeks 12 (visit 13) and 18 (visit 19), and used the correct method at both weeks 12 (visit 13) and 18 (visit 19), in accordance with the randomisation scheme. The safety outcomes were analysed in the safety set, comprising all patients who received ≥ 1 dose of rozanolixizumab treatment (partial or full). Additional outcomes were analysed in the randomised safety set, comprising all patients who were included in the safety set and were randomised. For continuous variables, the number of patients with available measurements, mean, standard deviation, median, and minimum and maximum were calculated; for categorical variables, the number and percentage of patients are presented. All analyses were performed using SAS Version 9.4 or higher.

## Results

### Patient population and exposure

**Fig. 2:**
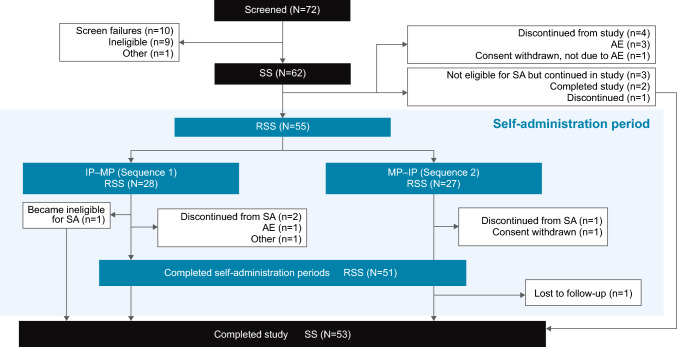
Patient study disposition. AE, adverse event; IP, infusion pump; MP, manual push; RSS, randomised safety set; SA, self-administration; SS, safety set

Overall, 62 patients received ≥ 1 dose of rozanolixizumab and comprised the safety set. Of this population, 55 patients were randomised, comprising the randomised safety set, to Sequence 1 (*n* = 28) or Sequence 2 (*n* = 27; Fig. 2). In the safety set, most patients had anti-AChR Ab+ gMG and were naïve to rozanolixizumab treatment at study entry (Table 1). In the randomised safety set, patient demographics and baseline characteristics were generally balanced between the two self-administration sequences. Overall, 48/62 (77.4%) patients in the safety set had a study medication exposure that spanned the entire treatment period (≥ 120 days). A post hoc analysis showed that in the randomised safety set, the median infusion duration across both Self-Administration Periods was 5 min (range: 1–30) with manual push and 12 min (range: 8–30) with infusion pump.

**Table 1:** Baseline demographics and patient characteristics

	Self-administration population (randomised safety set)			Overall population (safety set)
	Infusion pump–manual push (*N* = 28)	Manual push–infusion pump (*N* = 27)	RLZ total (*N* = 55)	RLZ total (*N* = 62)
Age, years, mean (SD)	52.9 (16.3)	53.4 (15.7)	53.1 (15.9)	53.3 (15.7)
Female, n (%)	16 (57.1)	15 (55.6)	31 (56.4)	35 (56.5)
*Weight, kg, n (%)*				
35 to < 50	1 (3.6)	0	1 (1.8)	1 (1.6)
50 to < 70	8 (28.6)	11 (40.7)	19 (34.5)	22 (35.5)
70 to < 100	15 (53.6)	11 (40.7)	26 (47.3)	30 (48.4)
≥ 100	4 (14.3)	5 (18.5)	9 (16.4)	9 (14.5)
BMI, kg/m^2^, mean (SD)	28.0 (5.7)	27.5 (5.4)	27.8 (5.5)	27.7 (5.4)
*Race, n (%)*				
Asian	1 (3.6)	3 (11.1)	4 (7.3)	5 (8.1)
Black or African American	1 (3.6)	0	1 (1.8)	1 (1.6)
White	25 (89.3)	24 (88.9)	49 (89.1)	55 (88.7)
Other/mixed	1 (3.6)	0	1 (1.8)	1 (1.6)
Age at initial MG diagnosis, years, mean (SD)	46.8 (18.9)	44.6 (18.4)	45.7 (18.5)	45.8 (18.3)
Duration of MG^a^, years, mean (SD)	6.4 (8.1)	9.4 (9.4)	7.9 (8.8)	7.9 (8.5)
Past myasthenic crisis, n (%)	8 (28.6)	6 (22.2)	14 (25.5)	15 (24.2)
Anti-AChR antibody-positive^b^, n (%)	21 (75.0)	20 (74.1)	41 (74.5)	46 (74.2)
Anti-MuSK antibody-positive^b^, n (%)	3 (10.7)	2 (7.4)	5 (9.1)	5 (8.1)
Anti-LRP-4 antibody-positive^b^, n (%)	0	0	0	1 (1.6)
RLZ-naïve at study entry^c^, n (%)	19 (67.9)	18 (66.7)	37 (67.3)	42 (67.7)
*Expected dose level, mg, n (%)*				
280	0	0	0	0
420	2 (7.1)	3 (11.1)	5 (9.1)	5 (8.1)
560	26 (92.9)	24 (88.9)	50 (90.9)	57 (91.9)
840	0	0	0	0
MG-ADL score, mean (SD)	7.1 (3.9)	7.5 (3.9)	7.3 (3.9)	7.3 (3.9)
*MGFA Disease Class, n (%)*				
Class I	1 (3.6)	0	1 (1.8)	1 (1.6)
Class IIa	8 (28.6)	6 (22.2)	14 (25.5)	17 (27.4)
Class IIb	4 (14.3)	6 (22.2)	10 (18.2)	11 (17.7)
Class IIIa	10 (35.7)	9 (33.3)	19 (34.5)	21 (33.9)
Class IIIb	5 (17.9)	6 (22.2)	11 (20.0)	12 (19.4)
Class IV–V	0	0	0	0
*Prior gMG medications*^*d*^*, n (%)*				
Parasympathomimetics	–	–	–	54 (87.1)
Corticosteroids	–	–	–	37 (59.7)
Immunosuppressants	–	–	–	31 (50.0)

Safety set and randomised safety set

*AChR*, acetylcholine receptor; *BMI*, body mass index; *gMG*, generalised myasthenia gravis; *kg*, kilogramme; *LRP-4*, low-density lipoprotein receptor-related protein 4; *m*, metre; *MG*, myasthenia gravis; *MG-ADL*, Myasthenia Gravis Activities of Daily Living; *MGFA*, Myasthenia Gravis Foundation of America; *MuSK*, muscle-specific tyrosine kinase; *RLZ*, rozanolixizumab; *SD*, standard deviation

^a^Duration of disease = (date of informed consent signed–date of initial MG diagnosis + 1)/365.25

^b^Historical autoantibody status; not reported for the remaining 16.1% of patients

^c^Patients were considered rozanolixizumab-naïve if they did not receive rozanolixizumab prior to study entry. All non-naïve patients participated in the MG0007 study

^d^Prior medications include any medications that started before the first administration of rozanolixizumab. Data for prior gMG medications were captured prior to the Training Period and were therefore analysed in the safety set

### Rate of successful self-administration

**Fig. 3:**
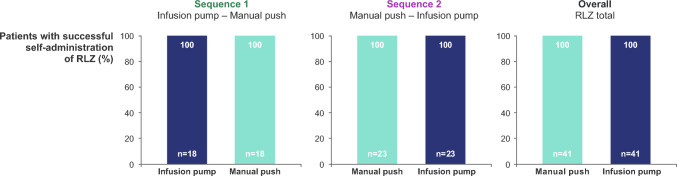
Self-administration success rate by study sequence. Full analysis set. Successful self-administration was assessed at week 12 (visit 13) for Self-Administration Period 1 and week 18 (visit 19) for Self-Administration Period 2. Percentages are based on the number of patients with non-missing data. Patients are grouped according to the self-administration method within the study period. RLZ, rozanolixizumab

All patients in the full analysis set (*n* = 41) had a 100% success rate for self-administration with both the infusion pump and manual push methods at weeks 12 and 18 (the last visit of Self-Administration Periods 1 and 2, respectively) (Fig. 3). There was also a 100% success rate for self-administration at all visits, both at home and at the study site, for all patients in the randomised safety set (Online resource 2).

### Safety

Overall, 47/62 (75.8%) patients who received ≥ 1 dose of rozanolixizumab reported TEAEs; most events were mild (138/165 [83.6%]) or moderate (23/165 [13.9%]) and incidence was comparable for both self-administration methods. The most frequently reported TEAEs overall were headache, coronavirus disease 2019 (COVID-19), pyrexia, diarrhoea, and nasopharyngitis (Table 2). Thirteen patients experienced a total of 29 headache events, of which most (21/29 [72.4%]) occurred during the first 6 weeks (during the Training Period), consistent with the known safety profile of rozanolixizumab. Moreover, events were generally reported early on within this 6-week period. Most events were mild (24/29 [82.8%]) and all resolved during the study, with 10/13 patients who experienced headache receiving oral analgesia. Most other TEAEs also occurred in the Training Period. TEAEs leading to permanent withdrawal of rozanolixizumab during the study were reported in 4 (6.5%) patients. These were one event each of metastatic lung cancer (serious and severe TEAE), MG worsening (serious TEAE) and migraine in individual patients, and pyrexia and headache in the same patient. These events all occurred during the Training Period, except for metastatic lung cancer. The events of migraine and headache were non-serious and reported in patients with a history of these events. Severe TEAEs were reported in 4 (6.5%) patients and were single cases of metastatic lung cancer (mentioned above), headache, MG worsening, and transient ischaemic attack. All severe TEAEs except for the event of headache were deemed unrelated to rozanolixizumab by the investigators. The patient with metastatic lung cancer was diagnosed during the study; they did not have a medical history of malignancy and were not receiving concomitant immunosuppressants or immunomodulators. The severe, non-serious headache event occurred during the Training Period after the first dose of rozanolixizumab; no other symptoms were reported. Treatment included concomitant non-opioid analgesics, and the event resolved after 9 days. Rozanolixizumab was temporarily discontinued, and when treatment was resumed the patient experienced mild events of headache only. Serious TEAEs were reported in 7 (11.3%) patients; the only serious TEAE reported in > 1 patient was MG worsening, which was reported in 2 (3.2%) patients and deemed unrelated to rozanolixizumab by the investigator in both instances. One event occurred during the Training Period and the other occurred during the Safety Follow-Up Period. One of the patients was rozanolixizumab-naïve and both patients were ADA negative. The observed incidence of TEAEs was consistent between the self-administration methods used and following the switch between the two methods. A post hoc analysis showed that the safety profile was generally consistent irrespective of infusion flow rate (≤ 20 mL/h versus > 20 mL/h).

**Table 2:** Overview of TEAEs

n (%)	Training Period (*N* = 62) (6 weeks)	Self-Administration Periods^a^ (*N* = 58) (12 weeks)	Safety Follow-Up Period^b^ (*N* = 53) (up to 7 weeks)	RLZ total (*N* = 62) (up to 25 weeks)
Any TEAEs^c^	35 (56.5)	30 (51.7)	6 (11.3)	47 (75.8)
Headache	12 (19.4)	4 (6.9)	0	13 (21.0)
COVID-19	4 (6.5)	2 (3.4)	0	7 (11.3)
Pyrexia	4 (6.5)	3 (5.2)	0	6 (9.7)
Diarrhoea	4 (6.5)	1 (1.7)	0	5 (8.1)
Nasopharyngitis	0	5 (8.6)	1 (1.9)	5 (8.1)
Serious TEAEs^d^	1 (1.6)	4 (6.9)	2 (3.8)	7 (11.3)
MG worsening	1 (1.6)	0	1 (1.9)	2 (3.2)
Permanent withdrawal of RLZ due to TEAEs	3 (4.8)	1 (1.7)	0	4 (6.5)
Treatment-related TEAEs^e^	19 (30.6)	8 (13.8)	2 (3.8)	22 (35.5)
Severe TEAEs	1 (1.6)	2 (3.4)	1 (1.9)	4 (6.5)
All deaths (AEs leading to death)^f^	0	0	0	0

Safety set

AE, adverse event; COVID-19, coronavirus disease 2019; MG, myasthenia gravis; RLZ, rozanolixizumab; TEAE, treatment-emergent adverse event

^a^Includes 55 randomised patients and 3 patients who were not randomised as they were not eligible for self-administration

^b^Patients who discontinued in the Training Period but completed the Safety Follow-Up are excluded

^c^Individual preferred terms listed under ‘Any TEAEs’ are those occurring in > 5% of patients in RLZ total

^d^Individual preferred terms listed under ‘Serious TEAEs’ are those occurring in > 1 patient in RLZ total

^e^Treatment-related was based on the investigators’ assessment

^f^All deaths are based on all patients screened and refers to all deaths occurring on study

No patients reported any local site reactions up to 24 h after administration, and no medication errors associated with adverse reactions were reported. Overall, in the safety set, 13/62 (21.0%) patients reported headache (Medical Dictionary for Regulatory Activities [MedDRA] high-level group term encompassing headache and migraine events). Most headache events (24/32) occurred in the Training Period. Twenty-six events were considered related to rozanolixizumab by the investigator, and 2/62 (3.2%) patients discontinued due to headaches. All events resolved with simple (non-opioid) analgesia. In total, 10/62 (16.1%) patients experienced gastrointestinal disturbance, 4/62 (6.5%) experienced hypersensitivity reactions, and 1/62 (1.6%) experienced an injection site reaction (1 day after the fourth infusion of the Training Period); all of these events were mild in intensity. Potential anaphylactic reactions associated with the use of rozanolixizumab were searched for using the MedDRA algorithmic Standardised MedDRA Query anaphylactic reaction and TEAEs that emerged either on the same day or one day after receiving rozanolixizumab. One patient reported simultaneous mild events of cough and pruritus that were reviewed and determined not to be an anaphylactic reaction. The events did not result in study discontinuation, and both resolved with administration of concomitant diphenhydramine hydrochloride, paracetamol, and benzonatate. A potential drug-related hepatic disorder was reported in one patient, with moderate single events of increased alanine aminotransferase, aspartate aminotransferase, and blood alkaline phosphatase. All events resolved and were deemed unrelated to rozanolixizumab by the investigator. There were no incidences of aseptic meningitis, opportunistic infections, effects on kidney, or TEAEs related to reductions in albumin or plasma proteins. The incidence of TEAEs related to the lipid panel was low, reported in 3 (4.8%) patients. No deaths were reported.

### Pharmacodynamics and immunogenicity

**Fig. 4:**
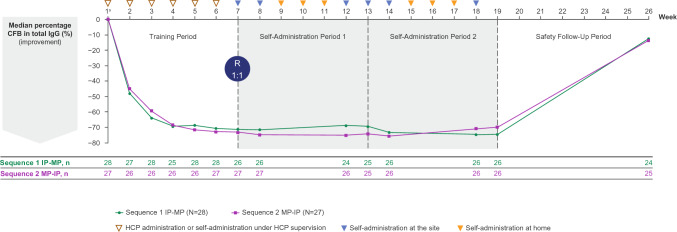
Median percentage change from baseline in total IgG. Randomised safety set. IgG values up to and including 8 weeks after the start date of rescue therapy were excluded. Median baseline value was 9.87 for Sequence 1 (*N* = 28) and 9.37 for Sequence 2 (*N* = 27). ^a^Baseline values were defined as the last available measurement before the first administration of study drug at week 1. CFB, change from baseline; HCP, healthcare professional; IgG, immunoglobulin G; IP, infusion pump; MP, manual push; R, randomisation

In the randomised safety set, there was a rapid reduction in total IgG within 1 week, with low levels sustained through to the end of the second Self-Administration Period. At week 2, median decrease from baseline in total IgG was 48.0% in Sequence 1 and 44.9% in Sequence 2 (Fig. 4). Median IgG levels decreased from week 1 by approximately 70% by the end of the Training Period and remained consistent over the 12 weeks of self-administration. No impact on IgG levels from switching self-administration methods was observed. The median maximum reduction from baseline was 74.6% (*n* = 26) and 75.6% (*n* = 26) in Sequences 1 and 2, respectively.

Overall, 27/53 (50.9%) patients developed treatment-emergent ADAs to rozanolixizumab. Five (9.4%) patients had pre-existing ADAs, of whom three demonstrated no significant change in ADA levels during the study (treatment-unaffected positive) whilst two exhibited an increase in ADA titres (treatment-boosted positive). All 5 patients had previously received rozanolixizumab in the MG0007 study. The presence of ADAs did not affect pharmacodynamic outcomes, with no evidence of a diminished or rebound effect on IgG levels, and no association was observed between ADA emergence and potential hypersensitivity or infusion site reactions.

### Efficacy

**Fig. 5:**
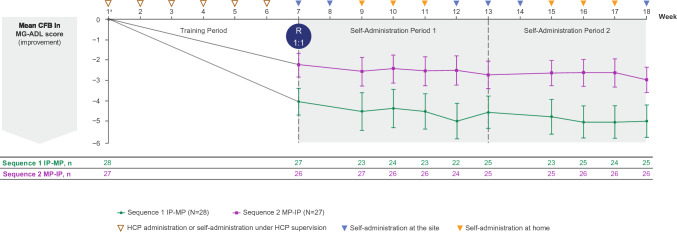
Mean change from baseline in MG-ADL score. Randomised safety set. Mean (SD) baseline value was 7.07 (3.89) for Sequence 1 (*N* = 28) and 7.52 (3.94) for Sequence 2 (*N* = 27). ^a^Baseline values were defined as the last available measurement before the first administration of study drug at week 1. CFB, change from baseline; HCP, healthcare professional; IP, infusion pump; MG-ADL, Myasthenia Gravis Activities of Daily Living; MP, manual push; R, randomisation; SD, standard deviation

Clinically meaningful improvements in MG-ADL score corresponding to ≥ 2-point improvement from baseline were observed in both sequences (Fig. 5). At the end of the Training Period (week 7), most patients experienced clinically meaningful improvement in MG-ADL. Improvements from baseline were consistent across the two Self-Administration Periods and no discernible impact on MG-ADL score from switching self-administration methods was observed. At the end of Self-Administration Period 2 (week 18), the mean change from baseline was –5.0 and –3.0 in Sequences 1 and 2, respectively.

A difference of approximately 2 points in mean MG-ADL score was observed between the two groups at week 7 and was preserved during the Self-Administration Periods. The difference in mean change from baseline between the two groups occurred purely by chance due to randomisation at week 7 resulting in a difference in the distribution of the changes from baseline in MG-ADL score at the end of the Training Period. The two patients with the largest improvement (≥ 12 points) during the Training Period were both randomly assigned to Sequence 1 and sustained this magnitude of effect throughout the Self-Administration Periods. Five of the six patients who demonstrated MG-ADL worsening during the Training Period were randomly assigned to Sequence 2, with all three patients who showed a ≥ 2-point worsening in MG-ADL allocated to Sequence 2 purely by chance.

### Patient preference and experience

**Fig. 6:**
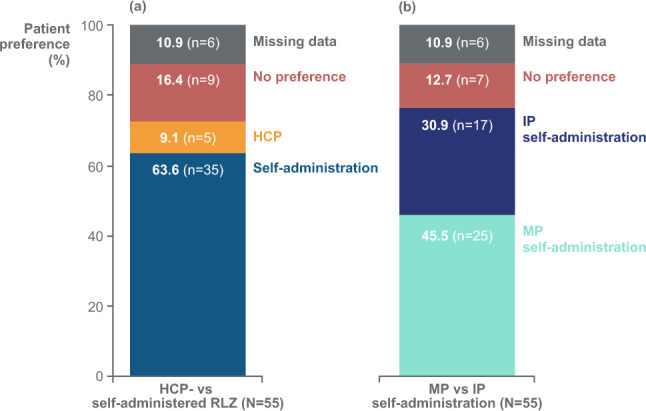
Patient preference for (**a**) HCP-administered versus self-administered rozanolixizumab and (**b**) manual push versus infusion pump self-administration**.** Randomised safety set. Questionnaire was administered at the last self-administration visit and answers were based on patients’ overall self-administration experience. Percentages are based on the number of patients with an assessment at week 18. Self-administration includes study drug administration by patient or caregiver. HCP, healthcare professional; IP, infusion pump, MP, manual push; RLZ, rozanolixizumab

The majority of patients (35/55 [63.6%]) preferred rozanolixizumab self-administration to administration by an HCP (5/55 [9.1%]), and more patients preferred the manual push method (25/55 [45.5%]) to the infusion pump method (17/55 [30.9%]) (Fig. 6). Most patients (41/55 [74.5%]) preferred self-administration at home, compared with only one patient who preferred administration in the hospital; a further seven patients did not have a preference, and data were missing for six patients. Preference was not influenced by the treatment sequence received.

Patients generally reported a positive experience with self-administration via both manual push and infusion pump methods. Mean pre-treatment SIAQ scores were 5.8–8.6; mean post-treatment SIAQ scores were 7.5–9.3, with a majority of patients scoring moderate to high satisfaction (Online resource 3). Domain scores were consistent between self-administration sequences, and following switching of administration methods all post-treatment SIAQ scores were > 7.3, demonstrating a consistently positive experience with self-administration using both methods.

## Discussion

Given the fluctuating nature of gMG symptoms and the high burden of care, broadening the administration options for rozanolixizumab would enable patients and caregivers to administer treatment in a manner aligned with their individual needs and preferences. Manual push and infusion pump methods are both well-established for the self-administration of SCIg; however, manual push is a simpler and more flexible method that has been preferred by patients with other diseases [22, 32]. This primary analysis of the phase 3 MG0020 study demonstrated that most patients with gMG were able to successfully self-administer rozanolixizumab, irrespective of self-administration method or site of administration (at home or in a hospital setting). Based on these results, in February 2025 and March 2025, the European Medicines Agency and the Japanese Pharmaceuticals and Medical Devices Agency, respectively, approved the self-administered treatment of rozanolixizumab via infusion pump or manual push method after training from an HCP [27, 29].

Self-administered rozanolixizumab was generally well tolerated, with a safety profile consistent with previous studies [24, 25, 33]. The most frequently reported TEAEs overall were headache, COVID-19, pyrexia, diarrhoea, and nasopharyngitis, with most TEAEs occurring in the Training Period and being of mild or moderate intensity. All headache events were mild or moderate except in one (1.6%) patient who reported a severe headache. MG worsening was the only serious TEAE reported in more than one patient. Importantly for self-administration, there were no medication errors associated with adverse reactions, and no patients had local site reactions up to 24 h after administration. There were no incidences of opportunistic infections, effects on kidneys, or TEAEs related to reductions in albumin or plasma proteins during the 18-week treatment period or the 7-week follow-up period. Incidence of TEAEs related to the lipid panel was low. Clinically relevant improvements were observed in MG-ADL score, consistent with the efficacy profile of rozanolixizumab reported in previous studies [24, 26, 33]. Although a difference in mean MG-ADL score of approximately 2 points was observed between the two treatment sequences during the Self-Administration Periods, the difference occurred purely by chance due to randomisation at the end of the Training Period resulting in a difference in the distribution of the changes from baseline in MG-ADL score. Rapid reductions and sustained low IgG levels were recorded within 1 week, in line with previous observations after rozanolixizumab treatment. Taken together, these data reassure patients and physicians that rozanolixizumab maintains its established safety, pharmacodynamic and efficacy profile regardless of administration type and method.

The majority of patients in the study had a positive experience with self-administration via both manual push and infusion pump methods, and preferred self-administration at home to HCP administration. These data support existing literature suggesting that patients with chronic immune diseases prefer self-administered SC treatment versus IV infusions for a number of reasons, including an increased sense of control and independence, reduced logistical and economic burden, and high treatment satisfaction [11, 17]. Preference for home administration has also been reported in patients with gMG; in a Patient Engagement Research Council composed of 13 patients with gMG and three caregivers, a preference for at-home and self-administered infusions was identified, with going to hospital described as a draining experience [34]. Similar clinical effectiveness has been demonstrated for SC and IV treatment in both immunological and autoimmune diseases [10, 35–37]; the most appropriate route of administration is likely to be determined by patient preference and individual circumstances [11]. Treatment satisfaction and HRQoL are also important factors to consider. Patients seeking greater autonomy and who are comfortable administering self-infusions are likely to be ideal candidates [19]. A higher proportion of patients preferred the manual push method to the infusion pump method, whilst post-treatment SIAQ scores indicated that patients had a positive experience with both methods. The manual push method is simpler for the patient as it requires less equipment [20, 21]; in addition, in MG0020, whilst both methods had a wide range for infusion duration, rozanolixizumab infusion duration was generally shorter with manual push than with the infusion pump method. Together, these factors may contribute to the observed patient preference for self-administration by the manual push method. As the MG treatment landscape continues to evolve, including SC administration in addition to traditional IV-administered therapies [3, 38], there is greater potential for patients to self-administer their treatment. Both self-administration methods would enable patients to be more independent, and would reduce costs for travel to clinic, infusion times, and absence from work required to receive treatment, amongst other benefits [5, 11, 17]. Considering the chronic and unpredictable nature of gMG, reducing patient burden is important to improve patients’ experiences of disease management [30, 39]. These findings support an alternative to HCP administration of rozanolixizumab and also favour manual push as a self-administration method based on patient preferences observed in this study.

This study has some limitations. There is a relatively small patient population and short treatment period, which may not have been sufficient to evaluate patients’ long-term preferences and treatment satisfaction; however, each patient in the randomised safety set performed 18 weekly infusions.

In conclusion, all patients who completed the two Self-Administration Periods in the MG0020 study successfully self-administered rozanolixizumab at all visits. Self-administration of rozanolixizumab via manual push was the preferred method, but a positive experience was reported with both methods. The safety profile, pharmacodynamics, and clinical response observed in this study were consistent with the known profile of HCP-administered rozanolixizumab, supporting self-administration and manual push administration as viable options for individuals with gMG.

## Supplementary Information

Below is the link to the electronic supplementary material.

Supplementary file1 (PDF 457 KB)

## Data Availability

Underlying data from this manuscript may be requested by qualified researchers 6 months after product approval in the US and/or Europe, or global development is discontinued, and 18 months after trial completion. Investigators may request access to anonymized individual patient-level data and redacted trial documents, which may include: analysis-ready datasets, study protocol, annotated case report form, statistical analysis plan, dataset specifications, and clinical study report. Prior to use of the data, proposals need to be approved by an independent review panel at www.vivli.org and a signed data-sharing agreement will need to be executed. All documents are available in English only, for a pre-specified time, typically 12 months, on a password-protected portal.
