# Supplementary material for: Self-administration of rozanolixizumab via manual push and infusion pump methods in patients with generalised myasthenia gravis: a randomised, phase 3, open-label, crossover study
Source: J Neurol. 2025 Oct 11;272(10):686. doi: 10.1007/s00415-025-13420-6 (PMC12515207; doi:10.1007/s00415-025-13420-6)
Supplement: Supplementary file 1 — Supplementary file1 (PDF 457 KB) [file 415_2025_13420_MOESM1_ESM.pdf]

Supplementary Information

Self-administration of rozanolixizumab via manual push and infusion pump methods in patients with generalised myasthenia gravis: A randomised, Phase 3, open-label, crossover study

Vera Bril[1], Carlo Antozzi[2], Tomasz Berkowicz[3], Artur Drużdż[4], Rachana K. Gandhi Mehta[5], Zabeen K. Mahuwala[6], Jana Zschüntzsch[7], Marion Boehnlein[8], Virginie Kerbusch[9], Andreea Lavrov[8], Mark Morris[10], Puneet Singh[10], M. Isabel Leite[11]

[1]Ellen and Martin Prosserman Centre for Neuromuscular Diseases, Toronto General Hospital, University of Toronto, Toronto, Ontario, Canada; [2]Neuroimmunology and Muscle Pathology Unit, Multiple Sclerosis Center, Fondazione Istituto di Ricovero e Cura a Carattere Scientifico, Istituto Nazionale Neurologico Carlo Besta, Milan, Italy; [3]Miejskie Centrum Medyczne Jonscher im. dr. Karola Jonschera w Łodzi, Łódź, Poland; [4]Department of Neurology, Municipal Hospital, Poznań, Poland; [5]Department of Neurology, Wake Forest University School of Medicine, Winston-Salem, NC, USA; [6]Department of Neuromuscular Medicine, Epilepsy and Clinical Neurophysiology, University of Kentucky, Lexington, KY, USA; [7]Department of Neurology, University Medical Center Göttingen, Göttingen, Germany; [8]UCB, Monheim, Germany; [9]PharmAspire BV, Wijchen, The Netherlands, ; [10]UCB, Slough, UK; [11]Nuffield Department of Clinical Neurosciences, University of Oxford, Oxford, UK

Corresponding author: Vera Bril, MD
Email: vera.bril@utoronto.ca

Online resource 1 Plain language summary

Generalised myasthenia gravis (gMG) is a chronic autoimmune disease that can cause extreme muscle weakness. In a previous study called MycarinG, treatment with rozanolixizumab once a week for six weeks improved disease symptoms versus placebo in adult patients with gMG. Based on the results of this study, rozanolixizumab was approved as a treatment for adults with gMG. Rozanolixizumab treatment was given by healthcare professionals (HCPs) as a subcutaneous injection (just under the skin) using a battery-powered infusion pump. We investigated options for patients to self-administer rozanolixizumab because it could increase convenience and patient satisfaction. The MG0020 study looked at whether patients could self-administer rozanolixizumab treatment using an infusion pump and a new method called manual push. The manual push method uses a hand-held syringe with a long thin plastic tube and can give the medicine more quickly than the infusion pump method. The manual push method also needs less equipment than the infusion pump method, so it may be easier for patients to use. Patients received rozanolixizumab treatment for a total of 18 weeks. This included a 6-week Training Period where patients learnt how to use the two self-administration methods, and two 6-week Self-Administration Periods where patients used one method and then switched to the other. Patients were allocated to one of two groups. The first group self-administered rozanolixizumab using the infusion pump method followed by the manual push method, and the second group self-administered rozanolixizumab using the manual push method followed by the infusion pump method. After the last dose in each Self-Administration Period, we looked at whether patients successfully gave themselves injections in the correct location, gave the injections just under the skin and gave themselves the correct dose.

In this study, 62 patients received rozanolixizumab treatment, of whom 55 completed both self-administration periods. Patients successfully administered all of their rozanolixizumab doses using both self-administration methods. Side effects were reported in 75.8% of patients. Most side effects were mild or moderate and the number reported was similar for

both self-administration methods. There were improvements in the Myasthenia Gravis Activities of Daily Living score compared to before starting rozanolixizumab treatment, suggesting that their gMG symptoms improved. These improvements were similar between the two treatment groups, suggesting that there was a similar effect for both self-administration methods and that changing the method had no impact. Most patients preferred self-administration of rozanolixizumab to administration by an HCP, and more patients preferred self-administration using the manual push method over the infusion pump method.

In this study, all patients successfully administered all of their rozanolixizumab doses using both self-administration methods. The preferred method of self-administration was manual push. Efficacy and safety were consistent with the results from previous studies where patients received HCP-administered rozanolixizumab.

Online resource 2 Self-administration success rate by site of administration

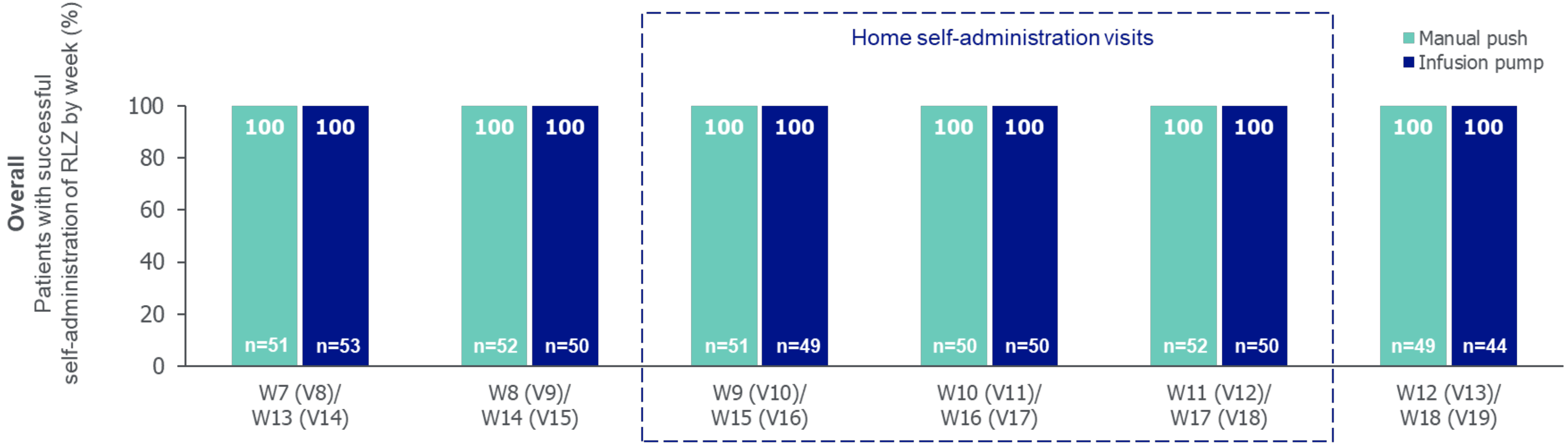

Patients with successful self-administration of RLZ by week, n (%)

		Self-Administration Period 1		Self-Administration Period 2		Overall	
Site	W7 (V8)/W13 (V14)	26 (100)	29 (100)	25 (100)	25 (100)	51 (100)	53 (100)
Site	W8 (V9)/W14 (V15)	27 (100)	24 (100)	25 (100)	26 (100)	52 (100)	50 (100)
**Home**	W9 (V10)/W15 (V16)	27 (100)	23 (100)	24 (100)	26 (100)	51 (100)	49 (100)
**Home**	W10 (V11)/W16 (V17)	27 (100)	25 (100)	23 (100)	25 (100)	50 (100)	50 (100)
**Home**	W11 (V12)/W17 (V18)	27 (100)	24 (100)	25 (100)	26 (100)	52 (100)	50 (100)
Site	W12 (V13)/W18 (V19)	29 (100)	20 (100)	25 (100)	26 (100)	49 (100)	44 (100)

Randomised safety set.

RLZ, rozanolixizumab; V, visit; W, week.

Online resource 3 Pre-treatment and post-treatment SIAQ score by domain

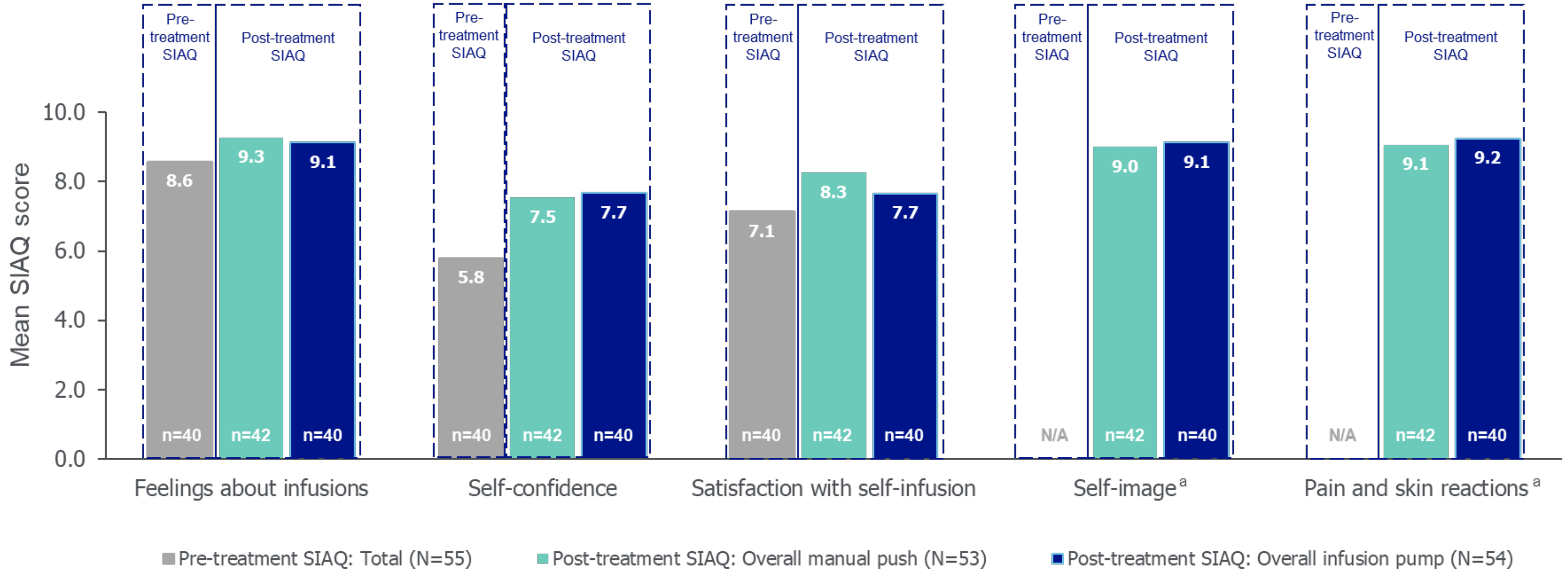

Randomised safety set. Domain scores were calculated if ≥50% of items in the domain were completed; domain scores range from 0–10, with a higher score indicating a better experience. Pre-treatment SIAQ was completed by patients pre-dose at Week 1, and post-treatment SIAQ was completed within 1 hour after self-infusion at Weeks 11 and 17. The SIAQ (Infusion Version) was not available in Georgia (n=6) and Serbia (n=2). [a]Self-image and pain and skin reaction domains were not included in pre-treatment SIAQ data.

N/A, non-applicable; SIAQ, Self-Injection Assessment Questionnaire.

Online resource 4 MG0020 co-investigators and contributors

Name	Location	Role	Contribution
**Akiyuki Uzawa**	Chiba University Hospital, Chiba, Japan	Principal Investigator	Supported with the acquisition of data
**Ali A. Habib**	ALS and Neuromuscular Center, University of California, Irvine, Orange, CA, USA	Principal Investigator	Supported with the acquisition of data
**Artur Drużdż**	Department of Neurology, Municipal Hospital, Poznań, Poland	Principal Investigator	Supported with the acquisition of data
**Carlo Antozzi**	Neuroimmunology and Muscle Pathology Unit, Multiple Sclerosis Center, Fondazione Istituto di Ricovero e Cura a Carattere Scientifico, Istituto Nazionale Neurologico Carlo Besta, Milan, Italy	Principal Investigator	Supported with the acquisition of data
**Denis Flemm**	UCB, Monheim, Germany	Operational study conduct	Supported with the acquisition of data
**Eugenia Martinez**	Hospital Clinic de Barcelona, Barcelona, Spain	Principal Investigator	Supported with the acquisition of data
**Franz Blaes**	Klinikum Oberberg - Kreiskrankenhaus Gummersbach - Klinik für Neurologie, Gummersbach, Germany	Principal Investigator	Supported with the acquisition of data
**Genya Watanabe**	National Hospital Organization - Sendai Medical Center, Sendai, Japan	Principal Investigator	Supported with the acquisition of data
**Gerardo Gutierrez**	Hospital Universitario Infanta Sofia Servicio de Neurología, Madrid, Spain	Principal Investigator	Supported with the acquisition of data
**Gordana Djordjevic**	University Clinical Centre Nis Clinic for Neurology, Nis, Serbia	Principal Investigator	Supported with the acquisition of data
**Jan Lünemann**	Universitätsklinikum Münster Klinik für Neurologie mit Institut für Translationale Neurologie, Münster, Germany	Principal Investigator	Supported with the acquisition of data
**Jana Zschüntzsch**	Universitätsmedizin Göttingen Klinik für Neurologie, Göttingen, Germany	Principal Investigator	Supported with the acquisition of data
**Ketevan Ugulava**	Israeli-Georgian Medical Research clinic Helsicore, Tbilisi, Georgia	Principal Investigator	Supported with the acquisition of data

**Kimiaki Utsugisawa**	General Hanamaki Hospital, Hanamaki, Japan	Principal Investigator	Supported with the acquisition of data
**Malgorzata Bilinska**	Uniwersyteckie Centrum Kliniczne, Klinika Neurologii Doroslych, Gdansk, Poland	Principal Investigator	Supported with the acquisition of data
**M. Isabel Leite**	Nuffield Department of Clinical Neurosciences, University of Oxford, Oxford, UK	Principal Investigator	Supported with the acquisition of data
**Marina Janelidze**	Simon Khechinashvili University Hospital, Tbilisi, Georgia	Principal Investigator	Supported with the acquisition of data
**Matteo Gastaldi**	Fondazione Istituto Neurologico Nazionale Casimiro Mondino- IRCCS - UO Neuroimmunologia, Pavia, Italy	Principal Investigator	Supported with the acquisition of data
**Min Kang**	University of California, San Francisco, San Fransico, CA, USA	Principal Investigator	Supported with the acquisition of data
**Phillip Ambrose**	Nottingham University Hospitals NHS Trust, Queens Medical Centre, Nottingham, UK	Principal Investigator	Supported with the acquisition of data
**Rachana K. Gandhi Mehta**	Department of Neurology, Wake Forest University School of Medicine, Winston-Salem, NC, USA	Principal Investigator	Supported with the acquisition of data
**Rual Juntas Morales**	Hospital Universitario Vall d'Hebron, Barcelona, Spain	Principal Investigator	Supported with the acquisition of data
**Roman Shakarishvili**	Petre Sarajishvili Institute of Neurology, Tbilisi, Georgia	Principal Investigator	Supported with the acquisition of data
**Shigeaki Suzuki**	Keio University Hospital, Toyko, Japan	Principal Investigator	Supported with the acquisition of data
**Stefania Morino**	Azienda Ospedaliera S. Andrea, UOC Neurologia, Rome, Italy	Principal Investigator	Supported with the acquisition of data
**Temur Margania**	New Hospitals, Tbilisi, Georgia	Principal Investigator	Supported with the acquisition of data
**Tomasz Berkowicz**	Miejskie Centrum Medyczne JONSCHER im. dr Karola Jonschera w Łodzi, Łódź, Poland	Principal Investigator	Supported with the acquisition of data
**Vera Bril**	Ellen and Martin Prosserman Centre for Neuromuscular Diseases, Toronto General Hospital, University of Toronto, Toronto, Ontario, Canada	Principal Investigator	Supported with the acquisition of data

**Yasushi Suzuki**	National Hospital Organization - Sendai Medical Center, Sendai, Japan	Principal Investigator	Supported with the acquisition of data
**Zabeen K. Mahuwala**	Department of Neuromuscular Medicine, Epilepsy and Clinical Neurophysiology, University of Kentucky, Lexington, KY, USA	Principal Investigator	Supported with the acquisition of data
